# Evaluation of a Bayesian hierarchical pharmacokinetic–pharmacodynamic model for predicting parasitological outcomes in Phase 2 studies of new antimalarial drugs

**DOI:** 10.1128/aac.00863-24

**Published:** 2024-08-13

**Authors:** Meg K. Tully, Saber Dini, Jennifer A. Flegg, James S. McCarthy, David J. Price, Julie A. Simpson

**Affiliations:** 1 Centre for Epidemiology and Biostatistics, Melbourne School of Population and Global Health, The University of Melbourne, Melbourne, Australia; 2 School of Mathematics and Statistics, The University of Melbourne, Melbourne, Australia; 3 Department of Infectious Diseases, The University of Melbourne, at the Peter Doherty Institute for Infection & Immunity, Melbourne, Australia; 4 Victorian Infectious Diseases Service, Royal Melbourne Hospital, Parkville, Victoria, Australia; 5 Walter and Eliza Hall Institute of Medical Research, Parkville, Victoria, Australia; 6 Nuffield Department of Medicine, University of Oxford, Oxford, United Kingdom; The Children's Hospital of Philadelphia, Philadelphia, Pennsylvania, USA

**Keywords:** pharmacokinetic–pharmacodynamic modeling, antimalarial, Bayesian methods, simulation

## Abstract

The rise of multidrug-resistant malaria requires accelerated development of novel antimalarial drugs. Pharmacokinetic–pharmacodynamic (PK-PD) models relate blood antimalarial drug concentrations with the parasite–time profile to inform dosing regimens. We performed a simulation study to assess the utility of a Bayesian hierarchical mechanistic PK-PD model for predicting parasite–time profiles for a Phase 2 study of a new antimalarial drug, cipargamin. We simulated cipargamin concentration- and malaria parasite-profiles based on a Phase 2 study of eight volunteers who received cipargamin 7 days after inoculation with malaria parasites. The cipargamin profiles were generated from a two-compartment PK model and parasite profiles from a previously published biologically informed PD model. One thousand PK-PD data sets of eight patients were simulated, following the sampling intervals of the Phase 2 study. The mechanistic PK-PD model was incorporated in a Bayesian hierarchical framework, and the parameters were estimated. Population PK model parameters describing absorption, distribution, and clearance were estimated with minimal bias (mean relative bias ranged from 1.7% to 8.4%). The PD model was fitted to the parasitaemia profiles in each simulated data set using the estimated PK parameters. Posterior predictive checks demonstrate that our PK-PD model adequately captures the simulated PD profiles. The bias of the estimated population average PD parameters was low–moderate in magnitude. This simulation study demonstrates the viability of our PK-PD model to predict parasitological outcomes in Phase 2 volunteer infection studies. This work will inform the dose–effect relationship of cipargamin, guiding decisions on dosing regimens to be evaluated in Phase 3 trials.

## INTRODUCTION

Almost 40% of the global population live in malaria endemic areas, with an estimated 249 million clinical cases in 2022, and over 608,000 deaths (1). Following a significant decrease in the global malaria burden between 2005 and 2015, the estimated number of malaria cases and deaths has begun to increase over the recent years (1). The availability of effective antimalarial drugs is key to reducing the burden of morbidity and mortality attributable to malaria.

Artemisinin-based combination therapies (ACTs), comprising a highly potent and rapid-acting artemisinin derivative with a longer-acting partner drug, are the current first-line treatment for *Plasmodium falciparum* malaria infection. However, partial resistance to artemisinins is now widespread across Southeast Asia (2) and more recently has emerged *de novo* in some African countries (3, 4), South America (5), and Papua New Guinea (6). Moreover, resistance to partner drugs used in ACTs, such as piperaquine, has also been detected in Southeast Asia (7), resulting in treatment failures. New antimalarial drugs are urgently needed.

Drug development is a resource-heavy, expensive, and time-consuming process, with only approximately 10% of drugs tested in Phase 1 trials ultimately gaining approval (8). The journey from early-phase clinical trials to Phase 3 clinical trials in patients, and then drug registration, can take many years (9). Cipargamin is a promising candidate antimalarial drug that has transitioned from early-phase studies (10) to Phase 2 clinical trials of adult patients with *P. falciparum* malaria (11, 12). In particular, it is a rapidly acting agent with potential to replace artemisinin (13). McCarthy et al. investigated the efficacy of cipargamin in a Phase 2 clinical trial (14) in eight healthy volunteer patients who were experimentally infected with malaria and 7 days later administered a low dose (10 mg) of cipargamin.

These human challenge studies, also known as volunteer infection studies, involve purposeful infection of healthy volunteers in a controlled environment and produce rich data on both parasite and drug concentrations through frequent sampling (15). Given the ethical considerations of infecting healthy volunteers, it is imperative that the maximum information possible is obtained from these data in order to guide selection of dosing regimens investigated for future Phase 2 and 3 studies. Statistical methods that are tailored to generating inferences from these valuable data are thus required. Pharmacokinetic–pharmacodynamic (PK-PD) modeling is a typical framework used for such analyses. These models integrate the PK model, which describes the drug concentration over time, with a PD model that characterizes the drug’s effect on the parasite population. Ideally, a PK-PD model should capture key elements of the underlying biological system, while remaining sufficiently simple for practical estimation and interpretation of key parameters (16).

In this study, we assessed an adaptation of an existing mechanistic Bayesian hierarchical PK-PD model developed by Dini et al. (17), which captures the life cycle of the parasite within the red blood cells. With a simulation-estimation framework, we investigated how precisely and accurately this model was able to recover the PK and PD parameters. The simulation study is based on data from the Phase 2 clinical study of cipargamin (14).

## RESULTS

**Fig 1:**
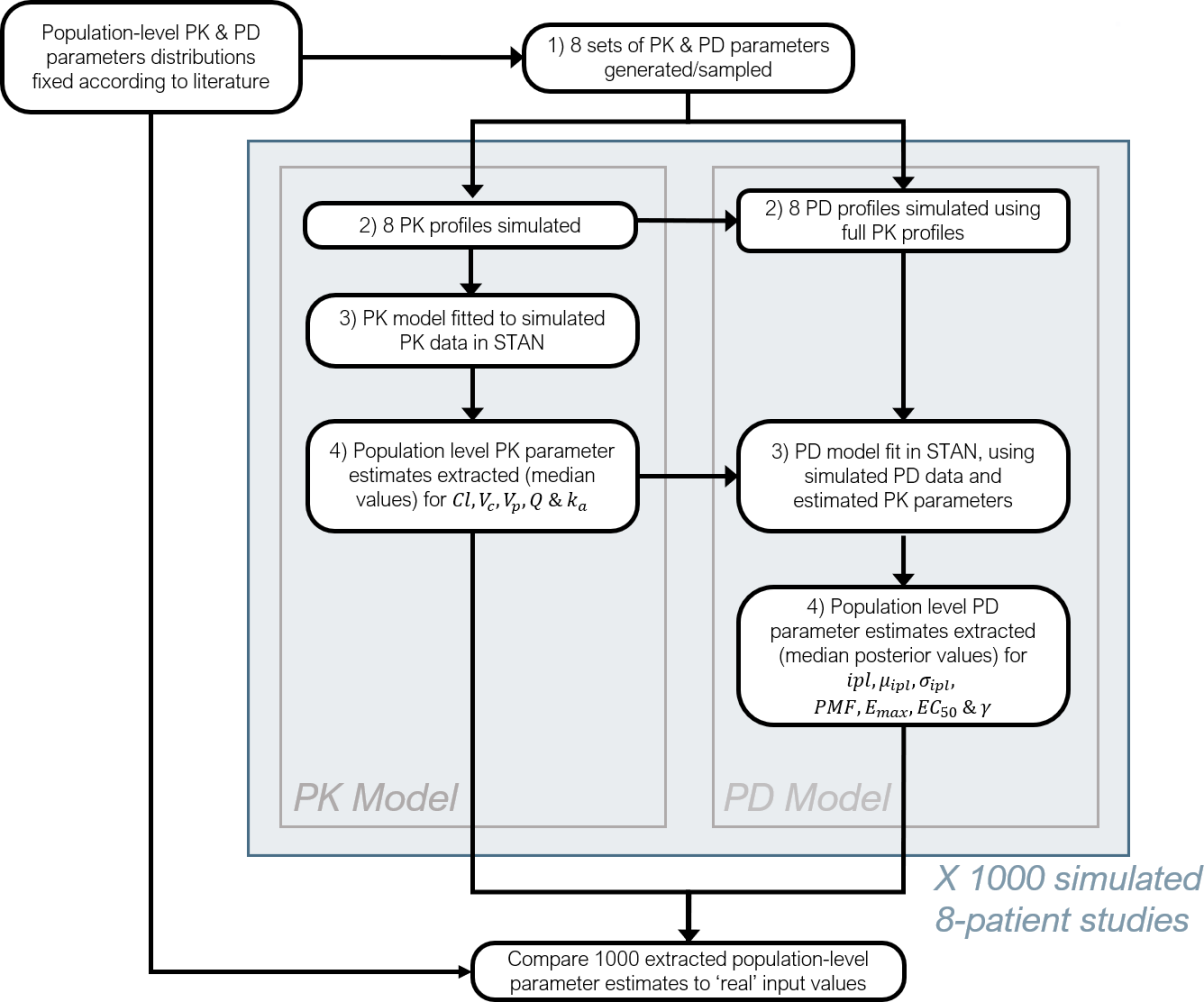
Flowchart describing the stages of the current simulation study framework.

A detailed description of the PK model, PD model, the Bayesian inference framework, and simulation study setup, including all model parameters, are provided in the *Methods* section. Definitions of the PK and PD model parameters are given in Tables 1 and 2, with a study overview diagram provided in Fig. 1.

**TABLE 1:** Definitions of pharmacokinetic model parameters

Parameter (units)	Definition
*Cl* (L/h)	Clearance rate of the drug
*V_c_* (L)	Central compartment volume
*Q* (L/h)	Inter-compartmental clearance rate
*V_p_* (L)	Peripheral compartment volume
*k_a_* (/h)	Absorption rate

**TABLE 2:** Definitions of pharmacodynamic model parameters

Parameter (units)	Definition
*ipl* (total #)	Initial parasite load. Total number of parasites at inoculation or model start
*µ_ipl_* (h)	Initial mean parasite age
*σ_ipl_* (h)	Standard deviation of the age distribution of the initial parasite load
*PMF*	Parasite multiplication factor. Number of parasites released by a ruptured schizont at the end of the life cycle
*E_max_* (% killed/h)	Maximal hourly killing rate of the drug
*EC*_50_ (ng/mL)	*In vivo* drug concentration when the killing rate is 50% of *E_max_*
*γ*	Slope of the *in vivo* drug concentration–effect curve

### Pharmacokinetic model

**Fig 2:**
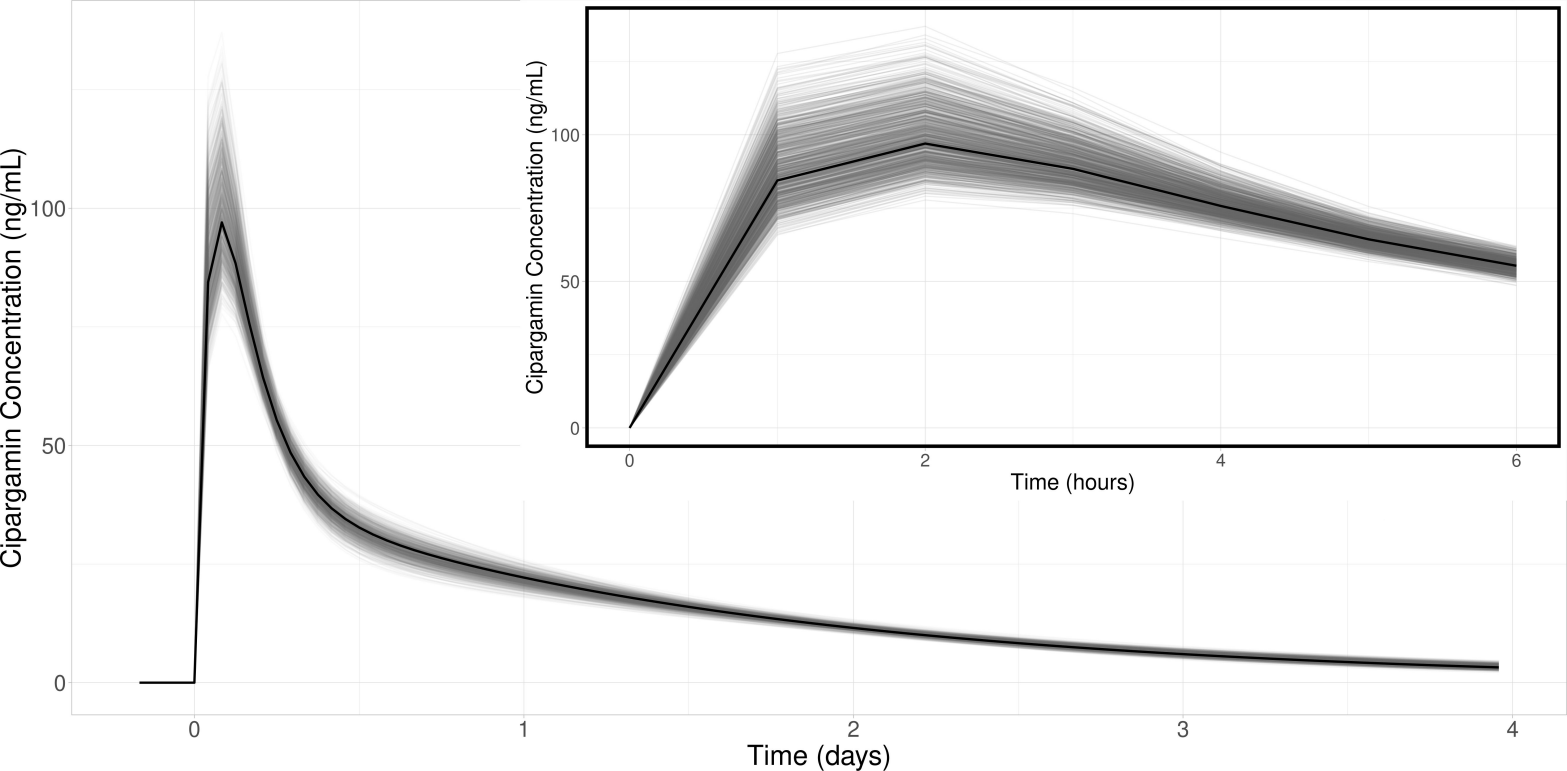
PK drug concentration profiles for a two-compartment model produced from “true” parameter values used in creating the simulations (black), compared to 1,000 profiles created from each of the 1,000 data set’s mean estimated values (gray).

Cipargamin concentrations were simulated using a two-compartment PK model with first-order absorption, based on the estimated PK parameters and between-individual variability from the analysis of the Phase 2 trial PK data (14) (Table 3). A total of 1,000 simulated data sets were generated, and each data set included the PK and PD profiles of eight patients, incorporating between- and within-individual variability. The simulated eight-patient PK data sets provided a good visual match to the trial data from McCarthy et al. (14) (Fig. S1). The PK model was incorporated into a Bayesian hierarchical framework and fitted to each of the 1,000 simulated data sets, restricting data to the cipargamin concentrations that correspond to the sampling times of the original Phase 2 trial (1, 2, 3, 4, 6, 8, 12, 16, 24, 36, 48, 72, 96, and 120 hours post-treatment), and the posterior median estimate of each population PK parameter was obtained. To evaluate how accurately this model can estimate PK parameters, we calculated the difference (absolute and relative bias) between the posterior median estimate of the population-level PK parameter and the value used to simulate the data (i.e., the “true” value). Table 4 shows the “true” PK parameter values used to simulate the data, the mean, 2.5- and 97.5-percentiles (herein, 95% intervals) across the 1,000 posterior median estimates associated with each simulation, and the bias (absolute and relative) in these posterior median estimates. The population-level PK parameters were reliably estimated, with the magnitude of relative bias ranging from 1.7% to 8.4%, comparing the mean of the posterior median estimates to the “true” value. To contextualize the bias in these estimates, we compared the PK profile created by the “true” population parameters to the PK profiles generated at the 1,000 posterior median parameter estimates (Fig. 2). This figure demonstrates that the average PK profiles for cipargamin are captured well across all simulations.

**TABLE 3:** Population parameters (***θ***) and feasible lower (***b***) and upper (***a***) prior bounds for each parameter in the first-order absorption two-compartment pharmacokinetic model for cipargamin

Parameter (units)	θ	[*b, a*]
*Cl* (L/h)	5.5	[2.75, 11]
*V_c_* (L)	64.4	[32.2, 128.8]
*Q* (L/h)	12.9	[6.45, 25.8]
*V_p_* (L)	107	[53.5, 214]
*k_a_* (/h)	0.919	[0.460, 1.838]

**TABLE 4:** Mean PK parameter estimates [95% intervals] over 1,000 fitted data sets and associated bias when compared to the values used to simulate the data^*a*^

Parameter (units)	“True” value	Posterior medians	Bias	
			Absolute	Relative (%)
*Cl* (L/h)	5.5	5.41 [5.08, 5.74]	-0.09 [-0.42, 0.24]	-1.64 [-7.64, 4.36]
*V_c_* (L)	64.4	61.89 [46.52, 77.84]	-2.51 [-17.88, 13.44]	-3.90 [-27.76, 20.87]
*Q* (L/h)	12.9	12.36 [10.12, 14.40]	-0.54 [-2.78, 1.50]	-4.19 [-21.55, 11.63]
*V_p_* (L)	107	111.44 [89.13, 139.44]	4.44 [-17.87, 32.44]	4.15 [-16.70, 30.32]
*k_a_* (/h)	0.919	0.996 [0.83, 1.21]	0.08 [-0.09, 0.29]	8.38 [-9.68, 31.66]

^*a*^ Estimates are the posterior median values from a Bayesian hierarchical model.

The population-level PK parameter least accurately estimated by the model was the absorption parameter, *k_a_*, with a mean relative bias of 8.4% [95% intervals (-9.7%, 32%)]. The PK profiles exhibit a short and sharp increase in drug concentration upon administration, during which absorption may be estimated; however, the availability of only one to two observations from this period impedes the estimation of the *k_a_* parameter. When the drug concentration profiles produced from the “actual” and “estimated” PK parameters were compared (Fig. 2), it is clear that the discrepancies between the absorption parameter values do not materially impact the cipargamin concentrations during the distribution and elimination phases.

**Fig 3:**
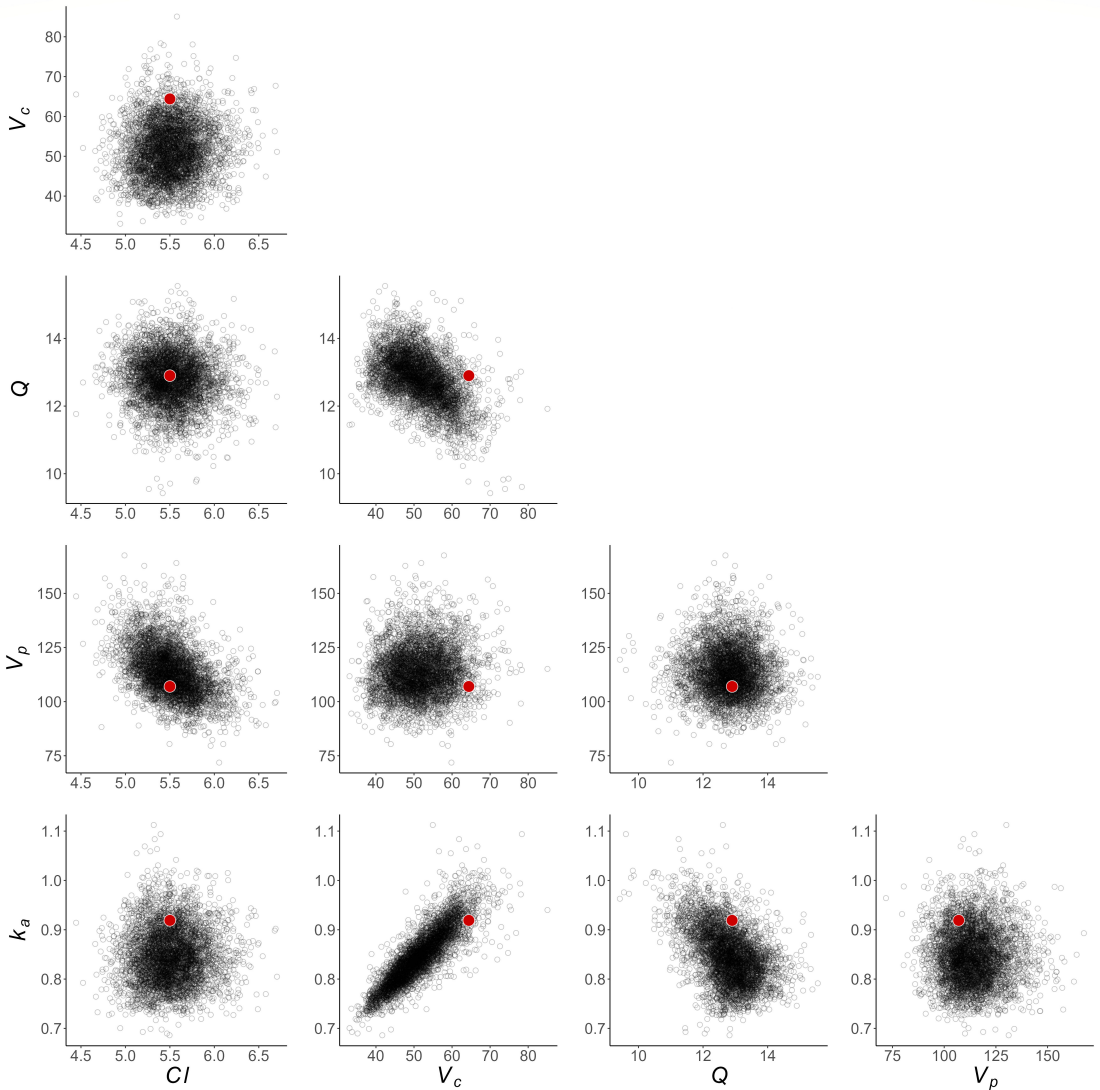
Bivariate distributions of posterior samples for population-level PK parameters, from the STAN fit of a single simulated data set. Red dots indicate “true” underlying parameter values used to simulate data.

To investigate how well this framework can recover model parameters for a single experiment, we show an example of the posterior samples compared to the “true” value in Fig. 3. These show that the true parameter values are contained within the range of posterior samples for each parameter, considering pairwise correlations. Figure S4 shows the posterior predictive pharmacokinetic profiles for each of the eight patients in a single experiment, again demonstrating that the posterior model fit provides an accurate characterization of the pharmacokinetic profile.

### Pharmacodynamic model

For each of the above simulated 1,000 data sets, the eight individual cipargamin concentration–time profiles were used to simulate eight parasite count profiles. These parasitaemia profiles were simulated for the initial 7 days of parasite growth post-inoculation. The simulated cipargamin concentration profiles were then used to simulate drug-induced killing of the parasites over the next 2 days, post cipargamin administration on day 7.

The PD model simulated the number of parasites aged 1 to 40 hours at each time point, and data for fitting the model were again restricted to the sampling times of the original study (72, 96, 108, 120, 132, 144, 156, 168, 172, 176, 180, 184, 192, 198, 204, 216, 228, 240, 264, and 288 hours post-innoculation). We assumed cipargamin had an immediate effect on the parasite and that the concentration–effect relationship followed Michaelis–Menten kinetics. The PD parameter values and feasible bounds selected for generation of the PD profiles are provided in Table 5. The 1,000 simulated PD data sets provided a good visual match to the parasitaemia data from the study by McCarthy et al. (14) (Fig. S6).

**TABLE 5:** Population parameters (***θ***) and feasible lower (***b***) and upper (***a***) prior bounds for each parameter in the pharmacodynamic model

Parameter (units)	θ	[*b, a*]
*ipl* (total #)	1800	[1500, 2100]
*µ_ipl_* (h)	2	[1, 24]
*σ_ipl_* (h)	3	[1, 14]
*PMF*	13	[5, 50]
*E_max_* (% killed/h)	0.23	[0.05, 1]
*EC*_50_ (ng/mL)	15.1	[0.5, 30]
*γ*	5	[1, 10]

**Fig 4:**
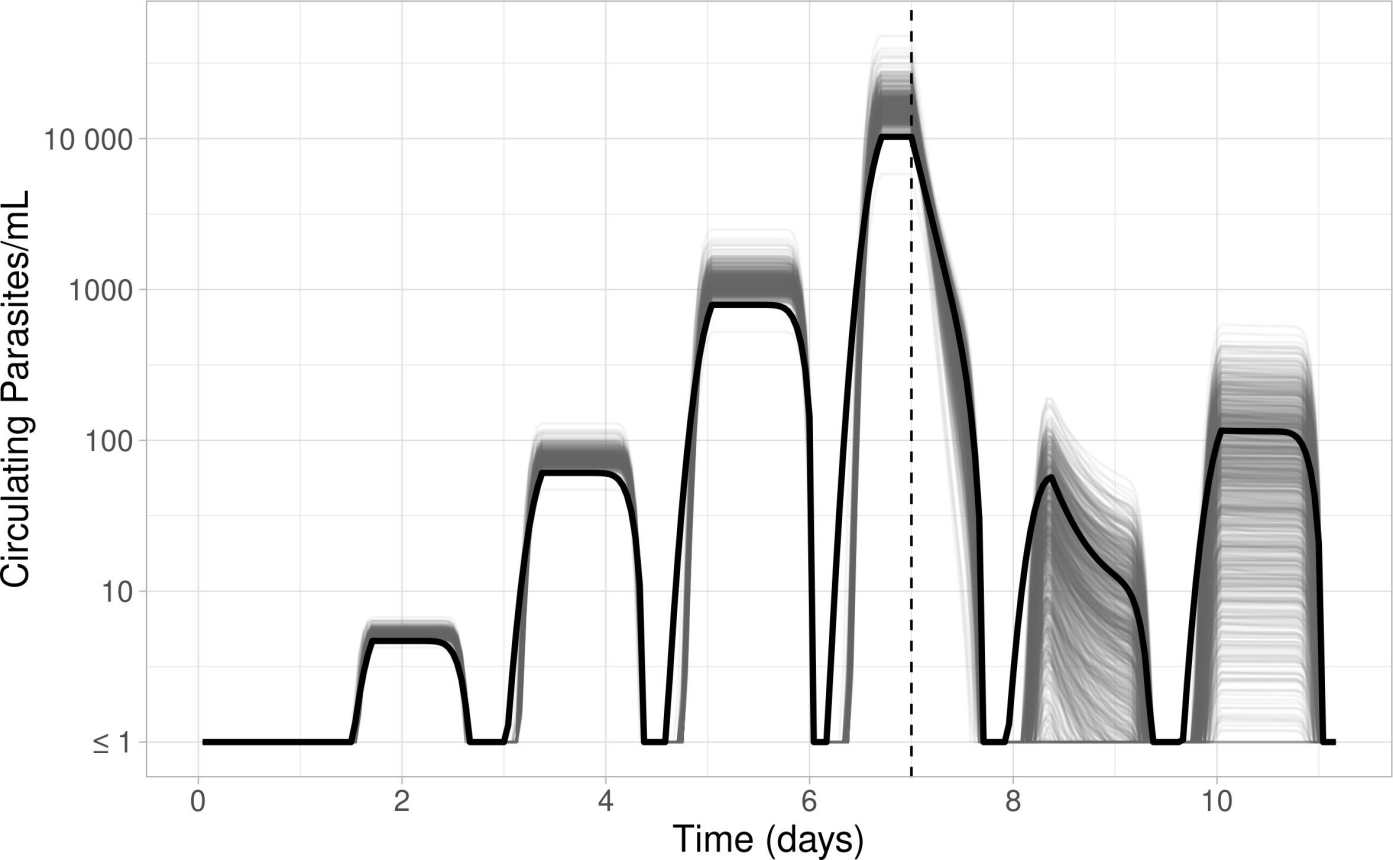
PD parasite profiles produced from “true” parameter values used to create the simulations (black), compared to 1,000 profiles created from each of the 1,000 data set’s mean estimated parameter values (gray). The dashed vertical line at day 7 indicates treatment.

Table 6 shows the “true” PD parameter values, the mean and 95% intervals across the 1,000 posterior median estimates, and the absolute and relative bias in the posterior median estimates for each PD parameter. The magnitude of relative bias for the posterior median estimates of the seven PD parameters varied between 1% and 53%. As per the PK evaluation, we contextualized this bias by plotting a profile produced by the mean PD parameter estimates for each of the 1,000 simulations and compared these to the PD profile created by the parameters used to simulate the data (Fig. 4).

**TABLE 6:** Mean PD parameter estimates [95% intervals] over 1,000 fitted data sets and associated bias when compared to the values used to simulate the data^*a*^

Parameter (units)	“True” value	Posterior medians	Bias	
			Absolute	Relative (%)
*ipl* (#*×*10^3^)	1.8	1.78 [1.75, 1.82]	-0.02 [-0.051, 0.018]	-1.11 [-2.83, 1.00]
*µ_ipl_* (h)	2	2.96 [2.69, 3.44]	0.96 [0.69, 1.44]	48.00 [34.50, 72.00]
*σ_ipl_* (h)	3	1.40 [1.28, 1.55]	-1.60 [-1.72, -1.45]	-53.33 [-57.33, -48.33]
*PMF*	13	14.55 [13.48, 16.80]	1.55 [0.48, 3.80]	11.92 [3.69, 29.23]
*Emax (% killed/h*)	0.23	0.29 [0.23, 0.38]	0.06 [0.00, 0.15]	26.09 [0.00, 65.22]
*EC*_50 (ng/mL)_	15.1	17.27 [13.80, 21.12]	2.17 [-1.30, 6.02]	14.37 [-8.61, 39.87]
*γ*	5	4.72 [2.86, 6.83]	-0.28 [-2.14, 1.83]	-5.60 [-42.80, 36.60]

^*a*^ Estimates are the posterior median values from a Bayesian hierarchical model.

The “true” mean initial parasite age (*µ_ipl_*) was 2 hours but had a mean estimate of 2.96 hours (95% quantiles: [2.69, 3.44]). Although there was a seemingly large relative bias (48% [34.50%, 72.0%]), this discrepancy is less than a 1-hour difference in parasite age. These still represent a mean age of parasites in the early ring stage of the parasite life cycle. Estimates of the standard deviation of the initial parasite age (*σ_ipl_*) are associated with a similarly large relative bias (-53.3%, [-57.3%,-48.3%]).

When we compared the profiles produced by the estimated and “true” values, (Fig. 4) the estimate-based profiles had slightly inflated pretreatment parasite counts due to overestimation of the PMF parameter. Bias in the estimation of the initial parasite age also resulted in the simulated profiles having approximately a 1-hour discrepancy in parasite trajectories. Overall, the bias in these estimates had a negligible impact on the general trajectory and parasite dynamics on a multi-day scale.

The estimated values of the PD parameters representing the maximum drug effect, *E_max_* (“true” value = 0.23), and the cipargamin concentrations at which half of this effect is achieved, *EC*_50_ (“true” value = 15.1), have a relatively moderate bias with mean posterior median estimates (95% quantiles) of 0.29 (0.23, 0.38) and 17.27 (13.80, 21.12) ng/mL, respectively. These estimates correspond to mean relative biases of 26.1% for *E_max_* and 14.4% for *EC*_50_. These PD parameters, together with *γ*, define the killing effect of the drug (Equation (2)). As a result, the bias in these estimates produces a noticeable discrepancy in the total number of parasites post-treatment (Fig. 4).

**Fig 5:**
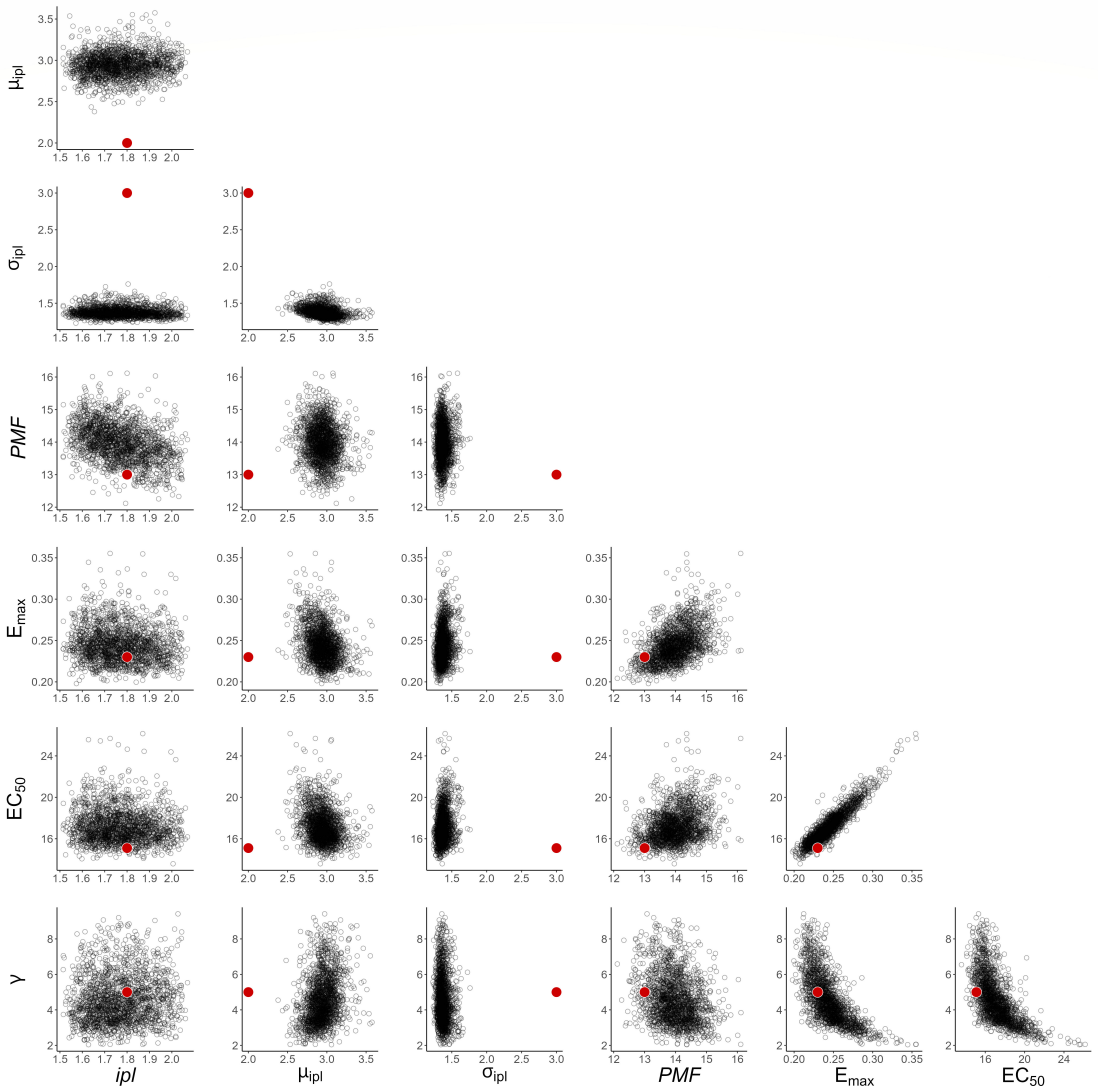
Bivariate distributions of posterior samples for population-level PD parameters, from the STAN fit of a single simulated data set. Red dots indicate “true” underlying parameter values used to simulate data.

As with the PK results, we demonstrate that this framework can recover PD model parameters (excluding the mean and spread of the initial parasite age distribution as described previously) for a single experiment by presenting an example of the posterior samples compared to the “true” value in Fig. 5. These show that, with the exception of *µ_ipl_* and *σ_ipl_*, the true parameter values are well-contained within the range of posterior samples, considering pairwise correlations. With the observed bias in the estimation of *µ_ipl_* and *σ_ipl_*, these posterior distributions exclude the “true” values entirely. Figure S7 shows the posterior predictive PD profiles for each of the eight patients in three randomly selected eight-patient cohorts, again demonstrating that the posterior model fit provides an accurate characterization of the PD profile.

## DISCUSSION

The results of this simulation–estimation study demonstrate that consequential parameters of the biologically informed PK-PD model can be estimated with moderate to high accuracy for Phase 2 volunteer infection studies, while parameters describing the initial distribution of parasite age are not estimated as well. The PK parameters in particular were all estimated with very low bias, whereas the estimation of certain PD parameters was less precise. The mean (*µ_ipl_*) and standard deviation (*σ_ipl_*) of the initial parasite age distribution corresponded to a relative bias of 48.0% and 53.3%, respectively. However, in absolute terms, this bias in the mean age corresponds to approximately 1 hour in the 40-hour parasite life cycle in patients observed over several days, which does not substantially impact the characterization of parasite dynamics. The *E_max_* and *EC*_50_ parameters had less relative bias (26.1% and 14.4%, respectively), yet these differences did lead to observable discrepancies in the post-treatment parasitaemia estimates for many of the simulated data sets.

Post-treatment parasite counts are often below the limit of quantification (LOQ). This model accounts for the measurement uncertainty in those data points by averaging across the range [0, LOQ], which provides some information on the relevant parameter values, but less than contributed by points measured above the LOQ. This imperfect observation contributes to the relatively poorer estimation performance of the PD model, and in particular, the bias in the estimates of the *E_max_* parameter. Generated data sets with more post-treatment observations under the LOQ had more biased parameter estimates and therefore poorer modeling accuracy (Fig. S8).

This form of the PK-PD Bayesian hierarchical model has been previously applied to volunteer infection and patient trial data sets (17, 18). The mechanistic form includes the hourly age of the parasite within the red blood cells for each individual, capturing the asexual reproduction cycle of the parasite and also allowing for the inclusion of the stage-specific action of the antimalarial drug. Estimates of the PK-PD model parameters can be derived using different statistical methods. Maximum likelihood methods are widely used in the analysis of data from early-phase antimalarial drug trials (9, 16). However, these methods have limitations that could potentially introduce significant bias and can fail to achieve convergence, especially for studies with small sample sizes, unless many of the parameter values are fixed. Additionally, the methods are restrictive in the incorporation of pre-existing data or knowledge. In contrast, Bayesian hierarchical methods have a number of advantages, such as explicitly incorporating prior knowledge and allowing for complex variation in both the population-level parameter values, and the correlations between the distributions from which patient-level values are drawn. Therefore, a direct empirical comparison of the two methods would not be a valid evaluation of their relative merit.

Pharmaceutical research and development is a costly and time-consuming process (19). Limited understanding of drug effects can result in the waste of resources though suboptimal trial design, simultaneously diverting efforts from other candidate treatments. Therefore, careful statistical analysis and interpretation serves to not only maximize the information obtained from a study but also has the capacity to reduce further inaccuracies, potentially limiting unnecessary risks for patients and minimizing delays in antimalarial drug development—and translation into practice. In addition, further computer simulation–estimation studies can be used to determine optimal sampling designs for future Phase 2 and 3 studies [e.g., (20, 21)].

Extrapolation and applicability of these simulation results is necessarily limited by the underlying assumptions of the simulation framework. This model is applied with the assumption that the underlying drug and parasite dynamics are identical to the form of the specified model. An area for further investigation would be evaluation of the impact of model misspecification on recovering biological parameters via a simulation–estimation study, whereby PK and/or PD dynamics are simulated under a different model to that used for fitting [e.g., (22)].

The model presented in this paper has been shown to reliably estimate key population-level PK–PD parameters within the sampling framework from a Phase 2 clinical trial of cipargamin (14), using simulated data. While some parameters are estimated with high bias, such as initial parasite age and spread, these differences are of small biological magnitude and did not materially impact the estimation of the drug effect. To date, there has been no published formal assessment in a simulation study of the ability of a Bayesian hierarchical PK–PD model to reliably estimate model parameters in the context of malaria. Therefore, this paper serves as an example of model performance evaluation through a simulation–estimation approach and provides confidence in the implementation of similar mechanistic malaria models and inference framework to analyze such data. This flexible model can be easily adapted to study and evaluate emerging antimalarial compounds in the future. By fitting this mechanistic PK–PD model to data from early-phase studies in a Bayesian framework, simulations from the resulting posterior distributions can inform the dosing schemes to evaluate in future phase 2 and 3 trials.

## MATERIALS AND METHODS

In this section, we describe the pharmacokinetic (PK) and pharmacodynamic (PD) models, the simulations generated from each, and the process of estimating model parameters from simulated data.

### Simulation of cipargamin pharmacokinetic profiles

This study simulated cipargamin concentrations using a standard two-compartment first-order absorption PK model with linear elimination (Text S3), as described in the study by McCarthy et al. (14). The definition of each PK model parameter is given in Table 1. A hierarchical (or mixed-effects) model was used to account for the between- and within-individual variability in cipargamin concentrations.

We simulated 1,000 data sets, each with PK profiles for eight patients, following the sampling intervals from the study by McCarthy et al. (14). Table 3 contains the population PK parameters, ***θ***, from the study by McCarthy et al. (14), and lower and upper bounds on each PK parameter. The bounds were chosen to allow a broad range of feasible values spanning half to double the PK estimates from McCarthy et al. (14). The prior posterior distribution comparison plots (Fig. S5) demonstrate the suitability of these ranges.

Multiplicative error terms for individual observations were drawn from a normal distribution with a mean of 0 and variance *σ*^2^ and then exponentiated. The *σ*^2^ value was generated individually for each data set, drawn from a log-normal distribution centered at 0.1 (see Text S4 for full details).

### Pharmacodynamic model

The PD model (presented and developed in (17, 23, 24) is a mechanistic representation of asexual parasite replication and death during the blood stage of the infection in the presence of an antimalarial drug, represented by a series of difference equations. Representing parasite age as an integer ranging from 1 to *T_max_*, the number of parasites that are *a* hours old at time *t*, *N* (*a, t*), is given by the number of parasites that were *a −* 1 hours old at time *t −* 1. The only unique case is the number of parasites that are 1 hour old at *t >* 0: this is given by the number of parasites that are at the end of the life cycle (*T_max_*) at the previous time step, *N* (*T_max_, t −* 1), multiplied by the parasite multiplication factor (PMF), representing the number of new merozoites released into the blood following the asexual reproduction of the parasite at the end of its life cycle. A stage-specific killing effect of cipargamin, *E*(*a, t*), at day 7 is then applied to the parasites of each age (Equation (1). Thus, the difference equations governing the parasite distribution are:


(1) $$N\left(a,t\right)=\left\{ \begin{array}{l} N\;\left(a\;-\;1,\;t\;-\;1\right)\times \;\left(1\;-\;E\left(a\;-\;1,\;t\;-\;1\right)\right),\;\;\;\;\;\;\;\;\;\;\;\;\;\;\;\;\;2\;\leq \;a\;\leq \;T_{max}\;\;\;\;\;\;\;\;\;\\ N\;\left(T_{max},\;t\;-\;1\right)\times \;\left(1\;-\;E\left(T_{max},\;t\;-\;1\right)\right)\times \;PMF,\;\;\;\;\;\;\;\;\;\;\;\;\;\;\;\;\;\;\;\;\;\;\;a\;=\;1.\;\;\;\;\;\;\;\;\; \end{array}\right.$$

Following inoculation, the initial age distribution, *N* (*a,* 0) is assumed to be normally distributed and discretized into hourly age groups. This distribution is defined by the number of parasites, *ipl*, and the mean, *µ_ipl_*, and standard deviation, *σ_ipl_*, of the parasite age distribution. During the growth phase, as the parasites age and replicate, the distribution shifts.

The effect of treatment on parasites of age *a* at time *t*, *E*(*a, t*), is assumed to have Michaelis–Menten kinetics and depend on the drug concentration *(C*(*t*)), the maximum killing effect (*E_max_*), the drug concentration for which 50% of that maximum killing effect is achieved (*EC*_50_), and the sigmoidicity of the concentration–effect curve (*γ*):


(2) $$E\left(a,\;t\right)\;=\;E_{max}\frac{C{\left(t\right)}^{\gamma }}{C{\left(t\right)}^{\gamma }+\;EC_{50}^{\gamma }},\;\;E\left(a,\;t\right)\;\;\in \;\left[0,\;1\right].$$

For this model, the life cycle was set to 40 hours in order to enable a visual match to the periodic trends of the trial data in McCarthy et al. (14), that were not reproducible with a 48-h cycle. This is consistent with Wockner et al. (25), where it was found that a range of 38.3 to 39.2 hours was the reproductive cycle length most strongly supported by their data from volunteer infection studies. Although Wockner et al. were using a different parasite dynamic model, these estimates were based on the same strain of malaria and a population of healthy volunteers with no prior malaria infections, similar to the participants of the trial data in McCarthy et al. (14).

### Simulation of parasite density versus time profiles

The 1,000 eight-patient parasite density-time data sets were simulated using the PD model, each corresponding to one set of simulated PK data. Each profile begins with a growth-phase starting from inoculation, followed by a treatment-phase from day 7 onward. The concentration profiles of the simulated PK data were input into the PD equation to generate the killing effect of the drug during treatment. Individual PD parameters were generated via the same approach as described for the PK parameters, that is, patient-level parameters were drawn from population-level distributions centered around ***θ***. Drug effect parameters were given by estimates from McCarthy et al. (14), and the parasite multiplication factor informed by (25). Table 5 contains the population PD parameters, ***θ***, and lower and upper bounds on each parameter. Aside from PK input data, the only other factors that varied between simulations were the variance–covariance matrix and noise distribution.

### Estimation of pharmacokinetic and pharmacodynamic parameters

For each of the 1,000 simulated data sets, parameters were estimated in a Bayesian framework using a Hamiltonian Monte Carlo No U-Turn Sampler in RStan v2.21.0 (26) using R version 4.1.1 (27). For fitting the PK model to the simulated cipargamin concentrations, three chains were run with 2,000 iterations each and 500 discarded as warm-up. This produced 4,500 posterior samples for each PK parameter, from which the posterior median was extracted as a central estimate of the posterior distribution.

*$\hat{R}$*, the effective sample size (*n_eff_*), trace plots, and posterior predictive interval plots were assessed to confirm that the chains had converged and were sufficiently well-mixed and that the posterior predictive distributions captured the simulated cipargamin concentration profiles accurately (Fig. S2 and S3).

For Bayesian modeling of the simulated parasitaemia data, three chains were run with 1,000 iterations each and 400 iterations discarded as warm-up, leaving 1,800 iterations for analysis. This was fewer than the number of iterations for each PK data set due to a comparatively longer processing time to evaluate the likelihood; however, visual assessment of the parameter trace plots confirmed adequacy of the burn-in period and suitable convergence. The same diagnostics were evaluated as for the PK model fitting in order to ensure chains were appropriately well-behaved, and posterior predictive distributions characterized the data (Fig. S7). See Supplemental text S5 for information on the prior distributions.

### Graphical representation

To evaluate the estimation accuracy of the PK-PD model, we compared the posterior medians to the “true” underlying input values. This comparison of the posteriors medians (mean [95% intervals]) is presented in Table 4 (for PK parameters) and 6 (PD parameters). Additionally, we plotted the hypothetical profiles that would be produced by each set of posterior median parameter values. These profiles are presented in Fig. 2 and 4 alongside the profile that would be produced by the true population values (i.e., centers of the population parameter distributions).

Fig. 3 and 5 present the full distribution of all posterior samples from the STAN fit of a randomly selected single data set.

All statistical computing codes for the simulation and estimation steps are available at https://github.com/M-Tully/pkpd_model_cip.

## SUPPLEMENTAL MATERIAL

Supplemental material aac.00863-24-s0001.pdf Pharmacokinetic/pharmacodynamic diagnostic checks, pharmacokinetic model, hierarchical simulations, and pharmacodynamic prior bound justifications.

ASM does not own the copyrights to Supplemental Material that may be linked to, or accessed through, an article. The authors have granted ASM a non-exclusive, world-wide license to publish the Supplemental Material files. Please contact the corresponding author directly for reuse.
